# Formation of Microcages from a Collagen Mimetic Peptide via Metal-Ligand Interactions

**DOI:** 10.3390/molecules26164888

**Published:** 2021-08-12

**Authors:** Jeremy Gleaton, Ryan W. Curtis, Jean Chmielewski

**Affiliations:** Department of Chemistry, Purdue University, 560 Oval Drive, West Lafayette, IN 47907, USA; jgleaton@purdue.edu (J.G.); rwcurtis@purdue.edu (R.W.C.)

**Keywords:** collagen mimetic peptide, hierarchical self-assembly, encapsulation, thermal release

## Abstract

Here, the hierarchical assembly of a collagen mimetic peptide (CMP) displaying four bipyridine moieties is described. The CMP was capable of forming triple helices followed by self-assembly into disks and domes. Treatment of these disks and domes with metal ions such as Fe(II), Cu(II), Zn(II), Co(II), and Ru(III) triggered the formation of microcages, and micron-sized cup-like structures. Mechanistic studies suggest that the formation of the microcages proceeds from the disks and domes in a metal-dependent fashion. Fluorescently-labeled dextrans were encapsulated within the cages and displayed a time-dependent release using thermal conditions.

## 1. Introduction

Collagen is a major structural protein found within a wide range of biological systems [1]. The ability to mimic the behavior of collagen is of high importance when considering its therapeutic value in the areas of regenerative medicine and drug delivery [2]. Attempts to manipulate natural collagen through physical and chemical means has been extensively explored and has resulted in the formation of a variety of higher-order structures such as microribbons [3], microspheres [4,5,6,7], and matrices for culturing cells in 3D [8], to name a few. The *de novo* design of collagen mimetic peptides (CMPs) containing additional higher-order assembly signals allows for the possibility of generating collagen peptide-based materials with morphologies and applications not found for natural collagen [9]. A variety of chemistries has been used to prompt the assembly of these peptides, such as exploitation of cysteine knots [10,11,12], native chemical ligation [13], electrostatic interactions [14,15,16,17,18,19], hydrophobic interactions [20,21,22], π-π interactions [23,24], and cation-π interactions [25]. 

The use of metal-ligand interactions as a means to prompt the assembly of CMPs has also been extensively explored [2,26,27]. The placement of ligands at the termini of triple helices has generated microflorettes [28,29], spiraled-horn networks [30], nanoropes [31], and petal-like structures [32,33] in the presence of metal ions, the latter of which demonstrate nanoscale banding, a behavior found within natural collagen. If ligands are incorporated at the center of the triple helix, stacked micro-sheets [34] or nanoscale disks [35] are formed after treatment with metal ions. Work with similar peptides demonstrates the formation of disks in the absence of metal, and addition of metal ions to these disks promotes hierarchical assembly into hollow spheres [36,37]. A marriage of both designs, wherein ligands are placed at both the center and termini of collagen peptides, yields the formation of a highly cross-linked morphology [38,39,40].

Previous work with the peptide **HByp3**, a CMP whose triple helix radially displayed nine bipyridine moieties, demonstrated self-assembly into micrometer-sized disks with a curved morphology [36]. These disks could further assemble into micron-sized hollow spheres upon addition of metal ions. Increasing the length of the triple helix by two repeat units of proline-hydroxyproline-glycine (POG) corroborated the mechanism for assembly of **HByp3** [37]. Thus, we postulated that increasing the number of bipyridine ligands displayed on a CMP would allow for a greater number of aromatic interactions between triple helices, and subsequent treatment with metal ions could result in the formation of higher-order structures. Herein, we describe the assembly of **HByp4**, a CMP containing four bipyridine moieties that forms triple helices. The hierarchical assembly of **HByp4** triple helices into disks and domes, followed by their assembly into micron-sized cages and cups after exposure to metal ions, is detailed, along with encapsulation and release of fluorescent cargo.

## 2. Materials and Methods

### 2.1. Materials

Fmoc-protected amino acids Fmoc-lysine(Mtt) (Mtt: 4-Methyltrityl), Fmoc-glycine-OH, Fmoc-proline-OH, and activation agent HBTU (O-benzotriazole-N,N,N′,N′-tetramethyl-uronium-hexafluoro-phosphate) were purchased from ChemPep, Inc. (Wellington, FL, USA). Fmoc-hydroxyproline(t-Bu)-OH and diisopropylethylamine (DIEA) was purchased from ChemImpex, Inc. (Wood Dale, IL, USA). ChemMatrix rink amide resin was purchased from pcas Biomatrix Inc. (Saint-Jean-sur-Richelieu, QC, Canada). Dichloromethane (DCM), N,N-dimethylformamide (DMF), methanol (MeOH), trifluoroacetic acid (TFA), triisopropylsilane (TIPS), and diethyl ether were purchased from Sigma-Aldrich (St. Louis, MO, USA). Fluorescently-labeled dextrans were purchased from Life Technologies (Grand Island, NY, USA). Congo Red was purchased from Enzo Life Sciences (Farmingdale, New York, NY, USA).

### 2.2. General Peptide Synthesis

**HByp4** was synthesized using standard 9-fluoromethylmethoxycarbonyl (Fmoc) protected amino acids on ChemMatrix Rink Amide resin (250 µmol, substitution = 0.52 mmol/g). Amino acids (6 eq, 1.56 mmol) were treated with HBTU (6 equiv, 1.56 mmol) and DIEA (12 equiv, 3.12 mmol) in DMF, the mixture was added to the resin, and this was agitated for 3 h at room temperature. After each coupling, the solution was drained and the resin was washed with DMF, DCM, MeOH, DCM, and DMF (2 × 10 mL). To remove the Fmoc protecting group, the resin-bound peptide was treated with piperidine (25% in DMF, 15 mL) for 30 min. The piperidine solution was drained and the resin was subsequently washed with DMF, DCM, MeOH, DCM, and DMF (2 × 10 mL). This process was repeated until the full length peptide was synthesized. After the final Fmoc deprotection, the N-terminal amino acid was acetylated using 5% acetic anhydride and 8.5% DIEA in DMF (*v*/*v*) for 30 min. The Mtt protecting groups were removed by treating the resin-bound peptide with 1.8% TFA in DCM (10 × 10 mL) with deprotection times of 3 min, with DCM washes (10 mL) in between each treatment. The resin was finally washed with DMF, DCM, MeOH, DCM, and DMF (2 × 10 mL), and a solution of 4′-methyl-2,2′-bipyridine-4-carboxylic acid (10 equiv, 2.6 mmol), HBTU (10 equiv, 2.6 mmol), and DIEA (20 equiv, 5.2 mmol) in DMF was added to the resin with agitation for 18 h. The resin was washed with DMF, DCM, MeOH, and DCM (2 × 10 mL), and was allowed to dry under reduced pressure. The peptide was subsequently cleaved from resin by the treatment with TFA/TIPS/H_2_O (95:2.5:2.5) (15 mL) (*v*/*v*) for 2 h. The TFA cocktail was drained into a round bottom flask, the resin was washed twice with TFA (15 mL) and twice with DCM (15 mL), and the collected filtrate solvents were removed under reduced pressures. This material was transferred to a conical tube and the peptide was precipitated with cold diethyl ether (50 mL). The precipitate was collected via centrifugation, and washed with additional cold diethyl ether (50 mL). The pellet was dried under flow of nitrogen, followed by drying under reduced pressure. The peptide was suspended in water/acetonitrile (10 mg/mL), followed by purification by reverse phase (RP) HPLC on a Luna C18 (250 × 21.20 mm, 100 Å pore size, 10 micron, Phenomenex) column. Eluent conditions consisted of solvent A (CH_3_CN/0.1% TFA) and solvent B (H_2_O/0.1% TFA) with a 60 min gradient of 10 to 40% solvent A, and a flow rate of 12 mL/min monitored at wavelengths of 214 and 254 nm. MALDI-TOF mass spectrometric measurements were performed to characterize the peptide: 3330.6 (calculated) 3330.1 (found). 

### 2.3. Circular Dichroism (CD)Spectroscopy

CD analysis was performed using a JASCO J-810 CD spectropolarimeter (Jasco Inc., Easton, MD) equipped with a PFD-425S Peltier temperature control unit. Prior to obtaining spectra, **HByp4** (150 µM) in buffer (10 mM HEPES, pH 7.0) was heated to 90 °C for 30 min. The peptide was then allowed to incubate at 4 °C for 24 h. Spectra were obtained at 4 °C with 3 scans between 210–300 nm at 0.1 nm data pitch and a 1 nm bandwidth. The scan rate was 100 nm/min with a 1 s response time. A CD melting curve was obtained by monitoring at 225 nm, with a temperature increase of 6 °C/h. The temperature range was 4 to 90 °C with a data pitch of 0.2 °C, and a bandwidth of 4 nm. The final melting temperature (T_m_) was determined by performing the first derivative (d[Ɵ]/dT) of this curve. 

### 2.4. Dynamic Light Scattering (DLS)

DLS measurements were performed on a Zetasizer Nano ZS (Malvern Instruments Ltd., Worcestershire, UK). The solutions were measured in plastic cuvettes and were placed in a sample holder at 22 °C. The intensity size distributions were obtained from the analysis of correlation functions using the multiple spherical modes algorithm. All solutions were filtered through a syringe filter having a pore size of 0.45 µm prior to the metal-ion treatment, incubation, and analysis.

### 2.5. Metal-Free Assembly into Disks and Domes

A solution of the peptide (250 µM) in HEPES buffer (10 mM, pH 7.0) was heated at 90 °C for 30 min, allowed to cool to room temperature, and stored at 4 °C for a total of 48 h. Prior to analyses, the samples were centrifuged at 10,000 g for 3 min and the supernatant was removed. The pellet was re-suspended in water (100 µL) and washed with water twice more.

### 2.6. Metal-Promoted Microcage Formation

A solution of the peptide (250 µM) in HEPES buffer (10 mM, pH 7.0) was heated at 90 °C for 30 min, followed by 6 h incubation at 4 °C. To this solution, the described metal ion (250 µM final concentration, dissolved in H_2_O) was added. For experiments with dextrans, the fluorescein-labeled 40 K dextrans (5 mg/mL, dissolved in water) were added to the solution prior to the addition of Fe(ClO_4_)_2_. All solutions were allowed to incubate for 48 h at 4 °C. Prior to the analyses, the samples were centrifuged at 10,000 g for 3 min and the supernatant was removed. The pellet was re-suspended in water (100 µL) and washed twice more. For samples stained with Congo Red, microcages were treated with Congo Red (500 µM final concentration, dissolved in H_2_O) for 24 h at 4 °C, followed by the washing protocol as described above.

### 2.7. Atomic Force Microscopy (AFM) 

A 5 µL aliquot of the sample was placed on freshly cleaved Mica surface (Ted Pella, Inc). This was allowed to air dry. The samples were then washed with 100 µL of water two times, with drying between each wash. Samples were imaged in air in tapping mode on a Multimode AFM with Nanoscope IIIa controller (Veeco) using oxide-sharpened silicon probes having a resonance frequency of 265–400 kHz (MikroMasch-NSC15, force constant: 40 N/m). All AFM images were obtained at room temperature.

### 2.8. Scanning Electron Microscopy (SEM) and Focused Ion Beam (FIB)

Samples were prepared by placing an aliquot of the material on the surface of a glass cover slip adhered to the specimen stub with a double-sided copper tape. The samples were allowed to air dry prior to being sputter coated with platinum for 60 s. Samples were imaged using an FEI NOVA nanoSEM field emission scanning electron microscope using the Everhart-Thornley detector (ETD) for low-resolution images or the high-resolution through-the-lens detector (TLD) to capture high-resolution images. Samples were imaged at an accelerating voltage of 5 kV with optimal working distances between 3–5 mm and a 30 µm aperture. For the focused ion beam analysis, the microcages were coated with a layer of platinum. The sample was tilted 52° and ablated with a 3 nA gallium ion beam source with a window of 18 × 7 × 5 µm. 

### 2.9. Cryogenic Scanning Electron Microscopy (SEM) 

Samples were transferred to a slit holder and plunged into a liquid nitrogen slush. A vacuum was applied and the sample was transferred to the pre-cooled (−160 °C) Gatan Alto 2500 pre-chamber. The sample was fractured with a cooled scalpel, producing a free-break surface. The sample was sublimed at −90 °C for 20 min followed by sputter coating with platinum for 120 s. To image, the sample was then transferred to the cryostage (−130 °C). These samples were imaged using an FEI NOVA nanoSEM field emission scanning electron microscope (FEI Company, Hillsboro, OR, USA) using the Everhart-Thornley detector (ETD) for low-resolution images or the high-resolution through the lens detector (TLD) for high-resolution images. The microscope was operated at an accelerating voltage of 5 kV, with optimized working distance and a 30 µm aperture.

### 2.10. Confocal Microscopy

Samples were imaged using a Nikon A1Rsi confocal microscope (Melville, NY, USA) with 488 and 561 nm laser lines for fluorescently-labeled dextrans and Congo Red, respectively. Samples were visualized on poly-L-lysine coated glass slides with a Nikon oil immersion 60× oil objective lens. 

### 2.11. Encapsulation and Release Experiments 

**HByp4** (250 µM) in HEPES (10 mM, pH 7.0) was heated at 90 °C for 30 min. Samples were then incubated for 6 h at 4 °C. Fluorescently-labeled dextrans were then added to the solution so that the final concentration of dextrans was 5 mg/mL. This was then vortexed for 30 s. After thorough mixing, Fe(ClO_4_)_2_ (250 µM) was added to this solution (final reaction volume 200 µL). These samples were allowed to incubate for 48 h at 4 °C. After incubating, the samples were spun at 4 °C at 10,000 g for 3 min. The supernatant was carefully removed and discarded. The pellets were then re-suspended in 500 µL of ultrapure Millipore water, centrifuged as above, and repeated twice. After the final wash with water, the supernatant was removed and 500 µL of phosphate buffer (pH 7.0, 10 mM) was added. The pellet was re-suspended followed by centrifugation at 10,000 g for 3 min at 4 °C. Again, the assemblies were washed twice with 500 µL of fresh phosphate buffer (10 mM, pH 7.0). On the final wash, the pellet was re-suspended in 200 µL of phosphate buffer (10 mM, pH 7.0).

A 50 µL aliquot of this solution was added to an Eppendorf containing 250 µL of phosphate buffer (10 mM, pH 7.0). This was again centrifuged at 10,000 g at 4 °C for 3 min. Next, 100 µL of the supernatant was transferred to a flat black bottom 96-well plate and the fluorescence of this solution was determined using a Tecan Spectra Fluor Plus microplate reader with excitation and emission wavelengths of 485 and 535 nm, respectively. After measuring the initial fluorescence ($F_{o}$), the solution was transferred back to its corresponding tube. The pellet was then completely re-suspended. It was then incubated at the described temperature for the described amount of time. The fluorescence at each time point was measured by centrifuging the sample at 10,000 g at 4 °C for 3 min and transferring 100 µL of the supernatant to the 96-well plate. After measuring the fluorescence value, the solution was transferred back to its corresponding tube to continue the experiment. 

At the conclusion of the experiment, samples were heated at 90 °C for 30 min. The solution was centrifuged at 10,000 g for 3 min at 4 °C. The supernatant was transferred to the 96-well plate and the fluorescence was measured. This measured number served as the 100% release value ($F_{100\%}$). The percent release (% Release) values at the previously described time points ($F_{t}$) were calculated as follows:

$$\%\;Release=\frac{\left(F_{t}-F_{o}\right)}{\left(F_{100\%}-F_{0}\right)}$$

The experiment was repeated twice in triplicate for each temperature studied.

## 3. Results and Discussion

### 3.1. Synthesis of HByp4 and Confirmation of Triple Helix Formation

**Figure 1:**
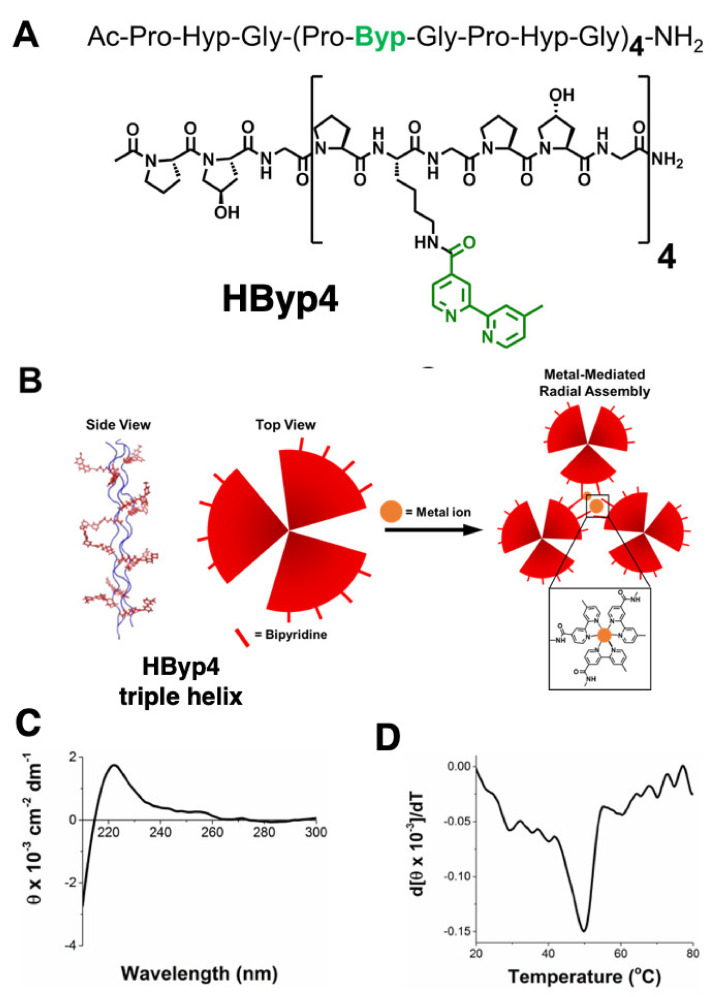
(**A**) The structure of HByp4; (**B**) metal ion-promoted assembly of the trimeric coiled coil; (**C**) CD spectrum of HByp4 (150 µM, 10 mM HEPES, pH 7.0); (**D**) first derivative of melting curve to determine the T_m_ value for HByp4.

HByp4 is a collagen mimetic peptide composed of Pro-Hyp-Gly repeats with the inclusion of four bipyridine moieties (Figure 1A). This peptide was designed to form a collagen triple helix displaying 12 bipyridine ligands, which would ultimately undergo higher order assembly upon the addition of metal ions through coordination with the ligands (Figure 1B). HByp4 was synthesized using previously described methods with standard solid phase peptide synthesis techniques [36,37]. Briefly, HByp4 was synthesized using the ChemMatrix Rink amide resin as a solid support with Fmoc-protected amino acids and HBTU with DIEA as the coupling reagent. Lys(Mtt)-OH residues were incorporated at the appropriate positions in the designed sequence to later incorporate bipyridine moieties (Figure 1A). Upon successful synthesis of the full-length peptide, the Mtt groups were removed under mild acidic conditions (1.8% trifluoroacetic acid in dichloromethane, 3 min), and 4′-dimethyl-2,2′-bipyridine-4-carboxylic acid was coupled to the free amino group using HBTU and DIEA. The peptide was subsequently cleaved from resin using a TFA cleavage cocktail (95:2.5:2.5 TFA/H_2_O/TIPS) and purified to homogeneity by reverse-phase HPLC and characterized with MALDI-TOF mass spectrometry.

Circular dichroism (CD) studies were performed to determine if the designed peptide adopted secondary structure characteristics matching those exhibited by collagen mimetic peptides. CD experiments showed that HByp4 displayed a maximum ellipticity at 225 nm, indicative of the presence of a polyproline type II helix (Figure 1C). Monitoring a decrease in the ellipticity at 225 nm as a function of temperature (4 to 90 °C), indicated that the designed peptide exhibited a cooperative dissociation, providing evidence for triple helix formation [41]. To determine the melting temperature (T_m_), a first derivative of the melting curve was performed (Figure 1D), and a T_m_ value of 50 °C was obtained for HByp4. This value constitutes a decrease in triple helix stability as compared to the parent peptide (Pro-Hyp-Gly)_9_ (T_m_ ~67 °C), which may be due to the replacement of hydroxyproline with the bipyridine-modified lysine residue. In CMP host-guest studies, replacement of this amino acid also led to triple helices with lower melting temperatures [42,43]. There was a modest increase in thermal stability relative to HByp3 (42 °C), which could be a result of the additional aromatic interactions facilitated by bipyridine groups within triple helices or as has been seen with other CMPs containing aromatic functionalities, CH∙∙∙π interactions of aromatic groups. In addition, proline residues could play a role in this observed additional stability [44].

### 3.2. Dynamic Light Scattering Analysis of HByp4

Previous studies with bipyridine-containing CMPs demonstrated that triple helices self-assemble into disks via bipyridyl interactions after thermal annealing [36,37]. Accordingly, HByp4 was thermally annealed in 10 mM HEPES, pH 7.0, and allowed to incubate at 4 °C for 48 h. Samples were then analyzed using dynamic light scattering (DLS). DLS data showed the solution to contain species with an average hydrodynamic diameter of ~2 µm. These assemblies are ~800 nm larger than those previously reported for HByp3, suggesting that HByp4 triple helices undergo additional assembly. To ascertain whether this assembly was a result of bipyridine interactions, HByp4 (250 µM) was thermally annealed under acidic conditions (10 mM glycine, pH 3.0) in order to protonate the bipyridine groups, which should limit the association between triple helices. After 48 h at 4 °C, the DLS analysis of this sample revealed a hydrodynamic diameter of 4.4 nm. This value is similar to previously reported individual triple helices of this length [36,45]. Dissolution of this morphology upon acidification of the ligands verified the crucial role of the bipyridine moieties in this assembly process.

### 3.3. Visualization of Assemblies Formed from HByp4 in the Absence of Metal 

**Figure 2:**
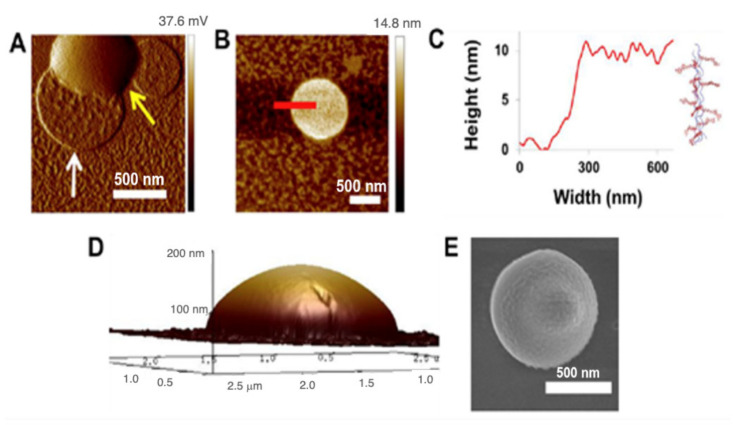
(**A**) A representative AFM amplitude image of HByp4 (250 μM, 10 mM HEPES, pH 7.0) after a 48 h incubation at 4 °C, white arrow–flatter disks, yellow arrow–domed structures; (**B**) AFM micrograph of the flatter disk morphology found in thermally annealed sample of HByp4 (250 μM, 10 mM HEPES, pH 7.0); (**C**) cross-section analysis of this disk shows a height of ~10 nm; (**D**) AFM topography map of the domed structures from (**A**) above; (**E**) cryo-SEM image of the dome structures. Scale bars: 500 nm.

To characterize the assembly that formed with HByp4, we visualized the material using AFM and SEM. HByp4 (250 µM, 10 mM HEPES, pH 7.0) was thermally annealed at 90 °C for 30 min followed by an incubation at 4 °C for 48 h. The white precipitate that formed was transferred to a freshly cleaved mica surface. The AFM analysis indicated the presence of round structures with diameters ranging from 500 nm to 1 µm (Figure 2A). Interestingly, two distinct morphologies were observed, a flatter structure (Figure 2A, indicated by a white arrow) and a “dome” structure (Figure 2A, indicated by a yellow arrow). The cross-section analysis of the flatter species showed that these structures have heights of ~10 nm (Figure 2B,C), a value consistent with the length of these collagen triple helices and a value previously reported for nanosheets and disks composed of CMPs [36,46,47]. These data suggest that HByp4 triple helices formed disks in a radial manner, with the triple helices aligned perpendicular to the surface with interactions between the 12 bipyridine groups of each of the triple helices with its neighbor. The protonation of the bipyridine groups in this assembly, as described above, reversed the assembly into individual triple helices.

The cross-section analysis of the AFM of the taller species, referred to as “domes” (Figure 2A, indicated by a yellow arrow), confirmed the rounded dome morphology (Figure 2D), and showed that the structures had heights that ranged from ~50 to 150 nm (determined from the analysis of eight different domes). To visualize the structures in a hydrated environment we enlisted the use of cryogenic scanning electron microscopy (Cryo-SEM). The cryo-SEM analysis complimented the findings with AFM and revealed round assemblies having similar diameters with an indication of similarity to the domed structures evidenced by the bump on the surface (Figure 2E). This increased height of the domes as compared to the disks, suggests that the four bipyridine moieties of HByp4 may promote additional interactions wherein some triple helices may not be completely buried within adjacent triple helices in the disks. This may lead to sticky ends on the disk surface that may allow for additional triple helical stacking on top of the disk. This added dimensionality during the assembly process may account for the formation of these taller domed architectures. With one less bipyridine group per CMP, HByp3 did not undergo this additional surface growth, and only formed the flatter disks.

### 3.4. Characterization and Visualization of Metal-Promoted Supramolecular Assembly of HByp4 

**Figure 3:**
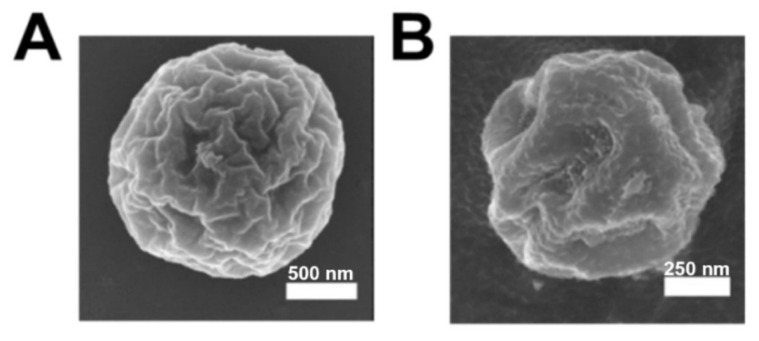
(**A**) SEM and (**B**) cryo-SEM of HByp4 (250 μM, 10 mM HEPES, pH 7.0) treated with Fe(II) (250 µM). Scale bars: 500 and 200 nm, respectively.

Since the self-assemblies described above still maintain ligands for metal ions, we next incubated the disks and domes of HByp4 with Fe(II), a metal ion capable of coordination with bipyridine ligands, to determine whether these structures would undergo metal-promoted assembly into higher-order structures. To investigate this, HByp4 (250 µM, 10 mM HEPES pH 7.0) was heated at 90 °C for 30 min and then incubated at 4 °C for 6 h to allow for disks/domes formation, followed by the addition of Fe(ClO_4_)_2_ (250 µM). This solution was allowed to incubate at 4 °C for 48 h, and the precipitate was then isolated and washed. The SEM analysis of the precipitate revealed the formation of spherical assemblies with wrinkled surfaces with sizes that ranged from 500 nm to 3 µm in diameter (Figure 3A). Visualization of the assemblies by cryo-SEM confirmed the spherical, puckered morphology in the hydrated state (Figure 3B).

**Figure 4:**
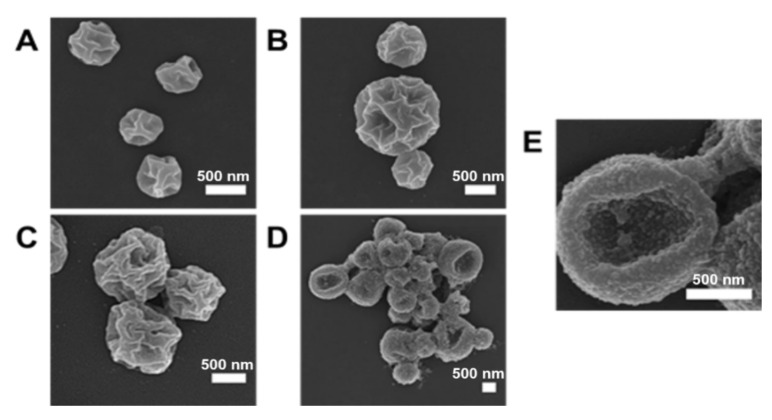
SEM micrographs for structures formed when a thermally annealed solution of HByp4 (250 µM, 10 mM HEPES pH 7.0) was treated with 250 µM of (**A**) Co(ClO_4_)_2_ (**B**) Cu(ClO_4_)_2_ (**C**) Zn(ClO_4_)_2_, and (**D**,**E**) RuCl_3_. Scale bars: 500 nm.

The metal-promoted assembly of the HByp4 disks and domes (250 µM) was also probed with Co(II), Cu(II), Zn(II), and Ru(III) (250 µM), as these metal ions are also capable of coordination with bipyridine. SEM visualization showed that the structures formed with Co(II), Cu(II), and Zn(II) resembled the puckered spheres observed with Fe(II), with slightly varying sizes (Co(II), 410–550 nm, *n* = 3. Cu(II), 670–1420 nm, *n* = 3. Zn(II), 890–1200 nm, *n* = 3) (Figure 4A–C). In the case of Ru(III) (Figure 4D,E), SEM micrographs revealed the presence of both puckered spheres and round, indented cup-like structures, the latter of which may be the result of the collapse of larger spheres.

**Figure 5:**
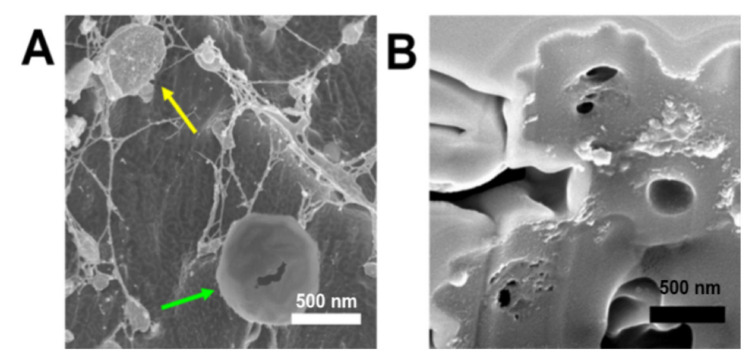
(**A**) Cryo-SEM micrograph of preformed HByp4-Fe(II) structures treated with EDTA (1 mM, 60 min). (**B**) SEM micrograph of HByp4-Fe(II) assemblies ablated with an ion beam. Scale bar: 500 nm.

Studies with the metal chelator, ethylenediaminetetraacetic acid (EDTA), were performed to help gain an understanding of the mechanism for metal-promoted assembly of HByp4. To this end, the HByp4-Fe(II) assemblies were treated with EDTA (1 mM) for 60 min, and this solution was visualized using cryo-SEM (Figure 5A). The cryo-SEM images showed the presence of the dome morphology reminiscent of those formed without metal ions (indicated by the yellow arrow). These micrographs suggest that the dome structures may be one component in the formation of HByp4 microcages. Partially disassembled microcages were also noted after the treatment with EDTA (Figure 5A, green arrow). These structures revealed a potential cavity hidden within the spherical assemblies.

To further probe the interior morphology, the spherical HByp4-Fe(II) structures were imaged using SEM in combination with a focused ion beam (FIB). This ablation with an ion source would allow for site-specific milling of these HByp4-Fe(II) structures. After the sample was milled, the stage was tilted, and the sample was imaged in a normal SEM mode (Figure 5B). These micrographs showed an exterior shell with an average thickness of 270 ± 95 nm (*n*=10), and the presence of a hollow interior that was ~200–300 nm in diameter. These ablation studies confirmed the formation of microcages with notable cavities. 

### 3.5. Encapsulation and Release of Dextrans from Microcages

**Figure 6:**
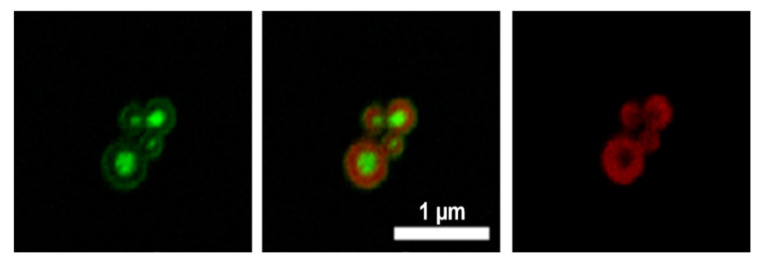
Representative confocal microscopy images for HByp4 with encapsulated 40 K MW dextrans. Fluorescein-labeled dextrans encapsulated mostly within the interior of the assemblies (**left**), with shells of the structures stained with Congo Red (**right**), and an overlap of both channels (**center**). Scale bar: 1 μm.

The identification of cavities with the HByp4-Fe(II) assemblies may allow for the inclusion of large cargo. In order to assess the ability of HByp4 microcages to encapsulate cargo, a 40 K fluorescein-labeled dextran (5 mg/mL) was added to a solution of thermally annealed HByp4 (250 µM) in HEPES (10 mM, pH 7.0) followed by the addition of Fe(II). After 48 h, the pellet that formed was collected and washed. These HByp4 assemblies were further stained with Congo Red (500 µM), a fluorophore that has been shown to bind to the triple helices of CMPs [28], washed, and visualized with confocal microscopy (Figure 6). These micrographs showed the shell of the cages labeled with Congo Red (Figure 6 right and center, red), and the encapsulated dextrans were mostly localized to the interior of the microcages (Figure 6 left and center, green), with a minor association of the dextrans with the exterior. 

**Figure 7:**
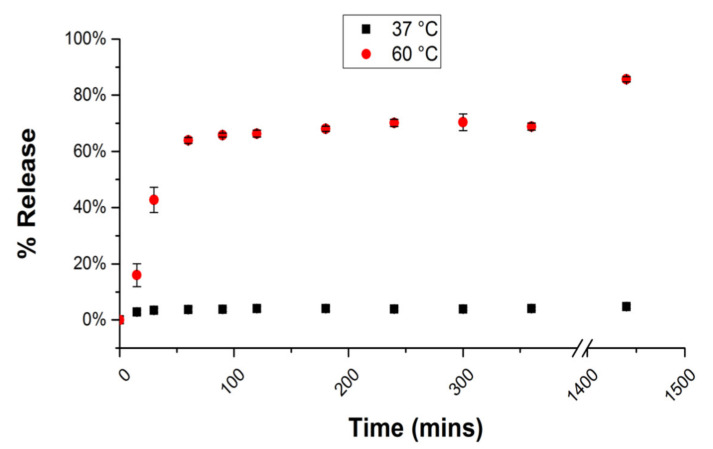
Release of fluorescein-labeled 40 K MW dextrans from HByp4-Fe(II) microcages at 37 and 60 °C in phosphate buffer (10 mM, pH 7.0) as monitored by the fluorescence analysis of the solution.

Since the building block of the microcages is the collagen triple helix, we envisioned that heating the sample may serve to disrupt the triple helices and the assemblies. Therefore, the release of the fluorescein-labeled 40 K dextrans from the HByp4-Fe(II) cages was monitored over a 24 h period at both 37 and 60 °C using the fluorescence of the solution. At the conclusion of 24 h, only ~10% of the dextran was released from the cages incubated at 37 °C. Increasing the temperature to 60 °C, however, afforded a marked increase in the release of dextrans, now displaying a release of ~90% after 24 h (Figure 7). These data demonstrate that heating can be an efficient strategy for cargo release from the CMP microcages.

## 4. Conclusions

Overall, the incorporation of four bipyridine units distributed along the length of a collagen mimetic peptide allowed for stable triple helix formation and subsequent assembly into nano- to micron-sized disks and domes, a condition that could be reversed by protonation of the bipyridine moieties. The addition of metal ions to these structures triggered supramolecular assembly into microcages through metal-ligand interactions, a process that could be reversed with the addition of a chelator for metal ions. These microcages were harnessed to encapsulate cargo, such as fluorescently-labeled dextrans, within the interior of the cages, a state that was reversed by heating the cages leading to the release of the cargo. CMP cages hold great promise for the encapsulation and delivery of cargo on demand, and future studies will explore incorporating other cargos, as well as tuning their rates of release.
